# DELIVER: Extending the benefits of SGLT-2 inhibitors

**DOI:** 10.21542/gcsp.2023.21

**Published:** 2023-08-01

**Authors:** Kerollos Samaan, Kerolos Wagdy

**Affiliations:** 1 Cardiology Department, Aswan Heart Centre, Aswan, Egypt; 2 Cardiology Department, Cairo University, Cairo, Egypt

## Abstract

The DELIVER trial investigated the efficacy and safety of dapagliflozin in patients with heart failure and preserved or mildly reduced ejection fraction. The trial demonstrated that dapagliflozin significantly reduced the risk of worsening heart failure or cardiovascular death compared to placebo. The benefit was mainly driven by a decrease in heart failure hospitalizations, with no significant impact on mortality. Patients with different ejection fractions and diabetes status showed similar treatment effects. Dapagliflozin also improved functional capacity and quality of life. These findings support the use of SGLT-2 inhibitors in HFpEF and HFmrEF, potentially influencing clinical practice and future guidelines.

## Introduction

Sodium glucose cotransporter-2 (SGLT-2) inhibitors were first introduced as a new therapy for type II diabetes mellitus. SGLT2 inhibitors have since gained an important role in the management of heart failure with reduced ejection fraction (HFrEF), particularly with the results of the DAPA-HF and EMPEROR-Reduced trials. Therefore, they have become one of the four pillars of HFrEF treatment, together with renin-angiotensin blockers, mineralocorticoid antagonists, and beta blockers^1,2^.

Patients with heart failure with mildly reduced ejection fraction (HFmrEF), preserved ejection fraction (HFpEF), improved ejection fraction (HFimpEF) still have limited data on their pharmacological options, due to a scarcity of trials addressing these groups of patients^3–5^.

Recently, the EMPEROR-Preserved trial studied the effect of empagliflozin in patients with an ejection fraction >40%. This showed a significant reduction in the combined endpoint of hospitalization for HF and cardiovascular death among these patients. In this trial, the benefit of reduced heart failure hospitalization was attenuated in higher left ventricular ejection fractions (LVEF ≥ 65%)^6^.

Dapagliflozin Evaluation to Improve the LIVEs of Patients with PReserved Ejection Fraction Heart Failure (DELIVER) trial^7^ aimed to assess the safety and efficacy of dapagliflozin in this group of patients, irrespective of their diabetes status.

Moreover, it has been designed to address some open questions, such as whether the benefits of SGLT-2 inhibitors are conserved in patients with the highest ejection fractions, and to determine the role of SGLT-2 inhibitors in a unique group of patients with HFimpEF (LVEF <40% then improved to >40%).

## The study

The DELIVER trial was a multicenter, double-blind, randomized controlled trial published in the New England Journal of Medicine (NEJM) in September 2022. The study enrolled 6263 patients with chronic heart failure with EF >40%, evidence of structural heart disease, and elevated natriuretic peptides. The patients were randomized in a 1:1 fashion into two groups: one arm (*N* = 3131) received dapagliflozin 10 mg once daily, while the other arm (*N* = 3132) received a placebo in addition to their usual therapy.

The patients were followed up for a median duration of 2.3 years for the primary endpoints of time to the first occurrence of hospitalization for worsening heart failure or cardiovascular death. The secondary outcomes included the total number of worsening heart failure events, cardiovascular deaths, changes in the total symptom scores in the Kansas City Cardiomyopathy Questionnaire (KCCQ; scores), and all-cause mortality.

The mean patient age was 71.7 years, 44% were females and 45% had type-2 diabetes mellitus. Regarding patient symptoms, 75% had New York Heart Association functional class II heart failure. The mean LVEF was 54% (≤49%: 34%; 50–59%: 36%).

**Figure 1.**
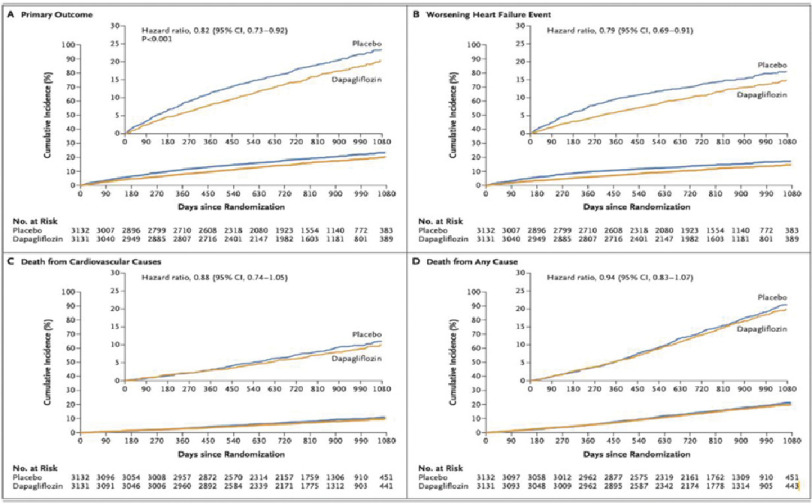
Primary and secondary outcomes of the DELIVER trial. .

The study showed a significant reduction in primary outcomes in the dapagliflozin arm (16.4%) compared to the placebo arm (19.5%) (hazard ratio, 0.82; 95% confidence interval [CI], 0.73 to 0.92; *p* < 0.001) (Figure 1A). The benefit was mainly derived from a reduction in hospitalization due to heart failure, not mortality (Figure 1B, C).

Regarding secondary outcomes, the total number of worsening heart failure events and cardiovascular deaths in the dapagliflozin arm (815) was significantly lower than that in the placebo arm (1057). Additionally, the change from baseline to month 8 in the KCCQ total symptom score indicated a benefit with dapagliflozin compared with placebo with respect to symptoms of heart failure.

However, there was an insignificant difference in all-cause mortality in both groups: 15.9% vs. 16.8% (HR 0.94, 95% CI [0.83–1.07], *p* > 0.05) (Figure 1D). The results were similar among patients with a left ventricular ejection fraction of >60% and those with a left ventricular ejection fraction of <60%, and the results were similar in patients with and without diabetes. The incidence of adverse events was similar between the two groups.

## Discussion

In this randomized, placebo-controlled trial involving patients with heart failure and a mildly reduced or preserved ejection fraction, dapagliflozin had a lower risk of the primary composite outcome, including worsening heart failure or cardiovascular death, than placebo did. This benefit was mainly derived from a reduction in the worsening of heart failure rather than mortality. Moreover, dapagliflozin improved the functional capacity and quality of life in this group of patients.

The EMPEROR-Preserved trial suggested some potential attenuation of benefit in the highest part of the range of the ejection fraction. In the DELIVER trial, the treatment effect was similar among patients with a left ventricular ejection fraction of ≥ 60% and those with a left ventricular ejection fraction of less than 60% with no evidence of heterogeneity. This finding suggests that the benefits of SGLT-2 inhibition are likely to extend throughout the full range of ejection fraction. The DELIVER trial also included patients who were hospitalized or recently hospitalized for heart failure (655 patients) and another group of patients with heart failure with improved ejection fraction (HFimpEF) (1151 patients), for whom evidence-based therapy is limited. The trial data suggest that both groups also benefit from SGLT-2 inhibitors.

## What have we learnt?

The DELIVER trial confirmed the extended benefits of SGLT-2 inhibitors in terms of reduced heart failure hospitalization and cardiovascular death in patients with HFmrEF and HFpEF, regardless of the presence of type 2 diabetes mellitus. The treatment effect is consistent in patients with LVEF>60% and patients with LVEF<60%. Its positive impact also presents a group of patients with heart failure with an improved ejection fraction. These findings may potentially affect the clinical practice and future guidelines for heart failure. Further prospective studies are warranted to determine the effects of SGLT-2 inhibitors on cardiac remodeling and hemodynamic function.
